# A plasmid-free *Zymomonas mobilis* mutant strain reducing reactive oxygen species for efficient bioethanol production using industrial effluent of xylose mother liquor

**DOI:** 10.3389/fbioe.2022.1110513

**Published:** 2022-12-23

**Authors:** Binan Geng, Shuyi Liu, Yunhao Chen, Yalun Wu, Yi Wang, Xuan Zhou, Han Li, Mian Li, Shihui Yang

**Affiliations:** ^1^ State Key Laboratory of Biocatalysis and Enzyme Engineering, Environmental Microbial Technology Center of Hubei Province, School of Life Sciences, Hubei University, Wuhan, China; ^2^ Zhejiang Huakang Pharmaceutical Co., Ltd., Quzhou, Zhejiang, China

**Keywords:** *Zymomonas mobilis*, plasmid-free, whole-genome resequencing (WGR), transcriptomics (RNA-Seq), proteomics, reactive oxygen species (ROS), oxyR, xylose mother liquor

## Abstract

Genome minimization is an effective way for industrial chassis development. In this study, *Zymomonas mobilis* ZMNP, a plasmid-free mutant strain of *Z. mobilis* ZM4 with four native plasmids deleted, was constructed using native type I-F CRISPR-Cas system. Cell growth of ZMNP under different temperatures and industrial effluent of xylose mother liquor were examined to investigate the impact of native plasmid removal. Despite ZMNP grew similarly as ZM4 under different temperatures, ZMNP had better xylose mother liquor utilization than ZM4. In addition, genomic, transcriptomic, and proteomic analyses were applied to unravel the molecular changes between ZM4 and ZMNP. Whole-genome resequencing result indicated that an S267P mutation in the C-terminal of OxyR, a peroxide-sensing transcriptional regulator, probably alters the transcription initiation of antioxidant genes for stress responses. Transcriptomic and proteomic studies illustrated that the reason that ZMNP utilized the toxic xylose mother liquor better than ZM4 was probably due to the upregulation of genes in ZMNP involving in stress responses as well as cysteine biosynthesis to accelerate the intracellular ROS detoxification and nucleic acid damage repair. This was further confirmed by lower ROS levels in ZMNP compared to ZM4 in different media supplemented with furfural or ethanol. The upregulation of stress response genes due to the OxyR mutation to accelerate ROS detoxification and DNA/RNA repair not only illustrates the underlying mechanism of the robustness of ZMNP in the toxic xylose mother liquor, but also provides an idea for the rational design of synthetic inhibitor-tolerant microorganisms for economic lignocellulosic biochemical production.

## Introduction

The production of sustainable, eco-friendly biofuels has become increasingly important in recent years due to the exhaustibility of fossil fuels and global climate change caused by excessive use of fossil fuels (Agarwal, 2007; Mills et al., 2009; Yang et al., 2018a). Bioethanol is an environmentally friendly and renewable liquid biofuel, and is one of the most promising alternatives to fossil fuels (Yang Y. et al., 2018). Lignocellulosic biomass is the most abundant, low-cost feedstock for bioethanol production (Balat et al., 2008; Zhang and Geng., 2012). However, these natural recalcitrant feedstocks are difficult to be directly utilized, therefore the processes of pretreatment and subsequent enzymatic hydrolysis are required to release sugars for microbial fermentation (Erdei et al., 2010; Yang Y., et al., 2018). During these processes, various toxic compounds such as acetic acid, furfural, HMF (5-hydroxymethyl-2-furaldehyde) are generated, which inhibit the substrate utilization, cell growth, and bioethanol production (Yang et al., 2018a). However, removal of these inhibitors isn’t economically feasible due to the increased cost caused by additional processing steps and the potential loss of sugars for fermentation during this process (Parawira and Tekere, 2011; Jonsson et al., 2013). Therefore, it is crucial to develop and employ inhibitor-tolerant robust microorganisms against inhibitory compounds within the lignocellulosic hydrolysates for economic bioethanol production from lignocellulosic materials.

Microbial genome reduction and modification are important strategies for constructing promising cellular chassis (Leprince et al., 2012). The removal of many non-essential regions from a bacterial genome conferred beneficial traits such as genotypic stability and phenotypic validity (Li et al., 2016). For example, *E. coli* strain MDS42 with a 663 kb region of the genome deleted displayed two orders of magnitude higher electroporation efficiencies (Posfai et al., 2006). The mutant strain *E. coli* DGF-298 had a more stable genome, improved growth rate and cell density after deleting a 1,670 kb region of the genome. For the production of bioproducts, the genome-reduction strain *B. subtilis* BSK814G2 (814.4 kb deleted) accumulated 115.2 mg/L of guanosine which was 4.4-fold increase than the control strain. and *B. subtilis* BSK756T3 (756.8 kb deleted) accumulated 151.2 mg/L thymidine, showing a 5.2-fold increase compared to the control strain (Li et al., 2016). Additionally, after deleting 7.7% of the genome of *P. mendocina* NK-01, the medium-chain-length polyhydroxyalkanoates (PHAMCL) and alginate oligosaccharides (AO) yields of the resulting strain NKU421 increased by 114.8% and 27.8%, respectively (Fan et al., 2020). Therefore, genome reduction could be a viable approach to obtain inhibitor-tolerant strains for efficient utilization of lignocellulosic hydrolysates.

*Zymomonas mobilis*, a non-model ethanologenic Gram-negative bacterium, has many desirable industrial characteristics. For example, *Z. mobilis* has a highly specific rate of sugar uptake, exhibits very high ethanol tolerance (16% v/v) and high ethanol productivity (5.67 g/g/h), and can grow under a broad range of growth temperature (24–45°C) and pH (4.0–8.0) (Wang et al., 2018; Yang et al., 2021). *Z. mobilis* strains naturally use the anaerobic Entner-Doudoroff (ED) pathway efficiently to consume glucose for the production of ethanol and other biochemicals such as gluconic acid, levan, and sorbitol (Erzinger and Vitolo, 1996; Silbir et al., 2014). In addition, gene editing tools have been continuously developed in *Z. mobilis* including exogenous and native CRISPR-Cas genome editing tools (Shen et al., 2019; Zheng et al., 2019), and *Z. mobilis* has become a promising chassis for the economic production of lignocellulosic biofuels and biochemicals such as 2,3-butanediol, isobutanol, and poly-3-hydroxybutyrate (PHB) (Yang et al., 2016; Qiu et al., 2020; Li et al., 2022).

Since the first transcriptomic and metabolomic study on the effects of oxygen on *Z. mobilis* fermentation (Yang et al., 2009b), transcriptomics has been widely used to study the stress responses of *Z. mobilis* to ethanol, furfural, acetate, phenolic compounds, and other inhibitors (He et al., 2012b; Yang et al., 2014b). It is also used to explain the molecular mechanism of mutants obtained through mutagenesis or adaptative laboratory evolution such as acidic-pH-tolerant mutants (Yang Q et al., 2020), and reveal metabolic pathway changes, such as the cysteine biosynthesis pathway after cysteine supplementation (Yan et al., 2022). In addition, proteomics strategy has also been applied to provide an in-depth understanding of the stress responses of ethanol, acetate, and other inhibitors in *Z. mobilis* (Yang et al., 2013; Yang et al., 2014b; Chang et al., 2018). Multi-omics approaches including transcriptomic, proteomic, and whole-genome resequencing have also been widely applied to investigate the changes and interrelationships of molecular components.

The genome of *Z. mobilis* model strain ZM4 includes a chromosome (2 Mb) and four native plasmids pZM32, pZM33, pZM36, and pZM39, which are 32–39 kb (Seo et al., 2005; Yang et al., 2009a; Yang et al., 2018b). These plasmids contain protein-coding genes involved in cellular defense, metabolism, and regulation (Yang et al., 2018b). For example, several genes involved in restriction and modification systems exist in pZM32. And genes encoding enzymes for metabolic functions are present in pZM33 including amidase, dehydrogenase, Acyl-CoA N-acyltransferase, NADPH-dependent oxidoreductase, and nucleoside triphosphate hydrolase. pZM36 contains several phage structure proteins. And genes encoding membrane-associated transporters, symporters, and porins are also identified in these plasmids, especially in pZM39. Addiction modules including toxin-antitoxin systems which have been reported to regulate stress adaptation and replicon persistence exist in all four plasmids (Unterholzner et al., 2013; Goeders & Van Melderen, 2014).

Although *Z. mobilis* is an excellent industrial microorganism for economic biochemical production from lignocellulosic materials, the capability of inhibitor tolerance can be further improved (He et al., 2012b; Yang et al., 2014a; Yi et al., 2015; Yang S et al., 2020). To examine whether the deletion of four native plasmids is beneficial for lignocellulosic ethanol production, we knocked out the cassette TetR-P*tet*-*cas12a* in ZMNP-Cas12a, a mutant with four native plasmids cured in previous work, to generate a plasmid-free mutant strain ZMNP in this study, and the ability of ZMNP to utilize lignocellulosic hydrolysate was further investigated.

## Materials and methods

### Strains, media, and culture conditions

*Escherichia coli* DH5α was cultured in Lysogeny Broth (LB, 10 g/L NaCl, 10 g/L tryptone, 5 g/L yeast extract) at 37°C with shaking at 220 rpm. *Z. mobilis* ZM4 (ATCC 31821) was cultured in Rich Medium (RM, 50 g/L glucose, 10 g/L yeast extract, 2 g/L KH_2_PO_4_) and Minimal Medium (MM, 50 g/L glucose, 1 g/L KH_2_PO_4_, 1 g/L K_2_HPO_4_, 1 g/L (NH_4_)_2_SO_4_, 0.5 g/L NaCl, 0.42 g/L MgSO_4_·7H_2_O, 0.001 g/L calcium pantothenate) at 30°C with shaking at 100 rpm. When required, 50 μg/ml of chloramphenicol was added to the LB and RM medium, respectively. The final concentration of furfural or ethanol added to the RM medium was 2.60 g/L (RMF) and 47.36 g/L (RME), respectively. The final concentration of furfural or ethanol added to the MM medium was 1.25 g/L (MMF) and 20 g/L (MME), respectively.

Xylose mother liquor used in this study was provided by Zhejiang Huakang Pharmaceutical Co., Ltd. (Kaihua, Zhejiang, China). The original hydrolysate contained 216.87 g/L glucose, 103.98 g/L xylose, 4.44 g/L furfural, and 1.18 g/L acetic acid. The hydrolysate was diluted with ×10 RM^−^ medium (100 g/L yeast extract, 20 g/L KH_2_PO_4_) for ethanol fermentation.

### Genetic manipulation and recombinant strain construction

Plasmid pL2R (Zheng et al., 2019) was used to knock out *cas12* gene in the chromosome. Meanwhile, gene *ZMO0038* was complemented at this position using the native Type I-F CRISPR-Cas system of *Z. mobilis* (Zheng et al., 2019). The spacer was 32 bp sequence which was immediately after a 5-NCC-3’ PAM in *cas12a*. The oligonucleotides (TsingKe, Beijing, China) of spacer were 36 bp with 4 bp protruding sequences in 5’-end. The editing plasmid was constructed following the previous description(Zheng et al., 2019). Briefly, the targeting gRNA sequence was constructed by annealed two single-stranded oligonucleotides (Cas12a-gR-F: 5’-gaa​atg​cgt​ttt​gaa​ctg​att​ccg​cag​ggt​aaa​acc, Cas12a-gR-R: 5’-gaa​cgg​ttt​tac​cct​gcg​gaa​tca​gtt​caa​aac​gca). Specifically, two single-stranded oligonucleotides were first heating at 95°C for 5 min and subsequently cooling down gradually to room temperature. Then the annealed spacer was ligated into *Bsa*I-linearized pL2R by T4 ligase at 22°C for 3 h. The resulting plasmid was named as pL2R-cas12a.

Gibson assembly method as described before (Li et al., 2022) was utilized for donor construction. Donor sequences including extra ∼800 bp upstream sequence and downstream sequence of the candidate gene were amplified using Primer STAR polymerase (Takara, Japan) from the genomic DNA of *Z. mobilis* ZM4. The upstream sequence was amplified using the oligonucleotide primer pair 0038-US-F (5’-ggt​cac​cag​ctc​acc​gtc​tgt​tag​gcg​aga​agg​gaa​agg​g) and 0038-US-R (5’-gtt​ggg​ttg​agc​cgc​gat​agt​cgt​taa​ata​ttc​aga​tag​acg​gag​ata​ata​aac​g), and the downstream sequence was amplified using the oligonucleotide primer pair 0038-DS-F (tcacgcccgacgccag) and 0038-DS-R (gct​cga​gat​ctg​ata​tca​ctc​acc​ctc​tgg​tga​ttg​tcg​at). The oligonucleotide primer pairs pL2R-FK-F (agt​gat​atc​aga​tct​cga​gct​cgg​tac​ccg​g) and pL2R-FK-R (aga​cgg​tga​gct​ggt​gac​ct) were used to amplify the pL2R-cas12a vector. The upstream sequence and downstream sequence were then cloned into pL2R-cas12a vector by T5 exonuclease (NEB, WA, United States). The resulting plasmids were named as pL2R-cas12aD. The editing plasmid pL2R-cas12aD verified by colony PCR and Sanger sequencing were transformed into *Z. mobilis* ZMNP-Cas12a to construct the final strain ZMNP.

### Electroporation transformation and recombinant strain selection

The editing plasmid pL2R-cas12aD was then transformed into *Z. mobilis* ZMNP-Cas12a competent cells (100 ng DNA with 50 μl competent cells) *via* electroporation using a Bio-Rad Gene Pulser (Bio-Rad, CA, United States). Immediately, the electroporated cells were transferred to 1 ml mating medium (50 g/L glucose, 10 g/L yeast extract, 5 g/L tryptone, 2.5 g/L (NH_4_)_2_SO_4_, 0.2 g/L K_2_HPO_4_, 1 mM MgSO_4_), and recovered at 30°C for 3 h. The cells were then spread on RM agar plates containing 50 μg/ml of chloramphenicol at 30°C for 2 days to isolate single colonies.

### Flask fermentation and analytic methods

For the seed culture preparation, glycerol stock solution of *Z. mobilis* strains ZM4 and ZMNP were inoculated into 5-ml RM medium and then cultured at 30°C. The cultures were then transformed into 200 ml RM medium which was in 250-ml flasks, and then cultured at 30 C without shaking. The seed culture of mid-log phase was then inoculated into 50-ml shake flasks containing 40 ml RM, MM, RMF, MMF, RME, MME medium, 1/3 xylose mother liquor with an initial OD_600_ value of 0.1. During the different time points of fermentation, the OD_600_ value of cell culture was determined by an ultraviolet spectrophotometer UV 1800 (AOE, Shanghai, China). Simultaneously, samples collected at different time points were centrifuged at ×12,000 g for 2 min, and then the supernatants were filtered through a 0.22-μm filters and stored at −80°C for measuring the concentrations of glucose and ethanol by HPLC analysis.

For HPLC analysis, HPLC (LC-20AD, Shimadzu, Japan) with an Aminex HPX-87H column (Bio-Rad, CA, United States) were used to measure the glucose and ethanol concentrations at 65°C. At the same time, 5 mM H_2_SO_4_ was as mobile phase at a flow rate of 0.5 ml/min.

### Whole-genome resequencing analysis

The sample ZMNP for whole-genome resequencing was collected and entrusted to GENEWIZ (Suzhou, China). The paired-end sequencing technology according to standard Illumina protocols by IgeneCode, Inc. (Beijing, China) was used in this work. The paired-end reads quality was checked using FastQC program (http://www.bioinformatics.babra.ham.ac.uk/projects/fastqc/). Data that passed the quality control were then mapped to the reference genome sequences of *Z. mobilis* ZM4 ATCC 31821 (GenBank accession No. of chromosome: NZ_CP023715, and plasmids: NZ_CP023716, NZ_CP023717, NZ_ CP023718, and NZ_CP023719) using the CLC Genomics Workbench (version 11.0) to identify the genomic variations. The objective mutations of the mutant strain ZMNP were obtained with the parental wild-type strain ZM4 as control. The mutation frequency which was more than 30% would be filtered.

### RNA-Seq transcriptomic analysis

The method of transcriptomic study is the same as reported previously (Yang et al., 2018b; He et al., 2018). Briefly, cell cultured under RM and MM medium were collected at the mid-log phase. Then total RNA extraction was using TRIzol reagent (Invitrogen, CA, United States). Then rRNA was removed from total RNA by using Ribo-off rRNA Depletion Kit (Bacteria NR407). Subsequently, mRNA was interrupted to short fragments by adding the fragmentation buffer. After synthesizing the first strand cDNA using random hexamer-primers, the second-strand cDNA was synthesized using buffer, dNTPs, RNase H and DNA polymerase I, respectively. The sequencing library were constructed by connecting fragments and sequencing adapters. And the transcriptome data were sequenced based on the Illumina NovaSeq 6000 System.

RNA-Seq fastq data which passed the quality control by FastQC program were imported into CLC Genomics Workbench (version 14.0) for reads trimming and RNA-Seq analysis to get the RPKM values of each gene with *Z. mobilis* ZM4 ATCC 31821 (GenBank accession No. of chromosome: NZ_CP023715, and plasmids: NZ_CP023716, NZ_CP023717, NZ_CP023718, NZ_CP023719) and four native plasmids as the reference genome. JMP Genomics (version 9.0) was used to normalize gene expression, analyze variance (ANOVA) and hierarchical clustering to identify differentially expressed genes at different conditions. Differentially expressed genes were determined with a selection threshold of *p*-value ≤ 0.01 and log_2_-fold change≥ ± 1 (significant induction).

### Protein sample preparation

The cells cultured in MM medium were collected and suspended into a 1 × RIPA buffer (×10: 150 mM NaCl, 10% NP40, 10% sodium deoxycholate, 10% SDS, 250 mM Tris–HCl, pH 7.6) at mid-log phase. Samples were grinded under low temperature for 5 min, and then ultrasonication on ice for 5 min. Subsequently, samples were kept at 4°C for 2 h and then centrifuged for 15 min at 4°C and ×12,000 g. 20 μl supernatant liquor containing extracted proteins of each sample was added into 96-well plate containing BCA buffer (Thermo Scientific, Rockford, United States). Then 96-well plate was shock at 37°C for 30 min, and the absorbance was detected at 562 nm (Thermo Scientific, CA, United States). The standard curve was then fitted, and the protein concentration of the corresponding sample is calculated. 100 μg of each sample was diluted to ∼1 mg/ml with 1 × RIPA lysis buffer.

Then each sample was precipitated by acetone overnight at −20°C. Subsequently, the protein precipitation was collected by centrifugation for 10 min at 4°C and ×12,000 g and then rewashed with pre-chilled 80% acetone two times. The protein precipitation was re-dissolved, reduced, and alkylated. The sequencing grade trypsin was then added at the ratio of 1:50 (wt:wt), and the digestion was run overnight at 37°C. The final protein precipitation was treated by sodium deoxycholate (SDC) cleanup and peptide desalting.

### Nano-LC-MS/MS analysis

1 μg total peptides of each sample were separated and analyzed with a nano-UPLC (EASY- nLC1200) coupled to a Q Exactive HFX Orbitrap instrument (Thermo Fisher Scientific, CA, United States) with a nano-electrospray ion source. A reversed phase column (100 μm ID ×15 cm, Reprosil Pur 120 C18-AQ, 1.9 μm, Dr. Maisch) was used to separate different sizes of peptides. Mobile phases were H_2_O with 0.1% FA, 2% ACN (phase A) and 80% ACN, 0.1% FA (phase B). Each ample was separated by executed with a 120 min gradient at 300 nL/min flow rate. Gradient B: 2%–5% for 2 min, 5%–22% for 88 min, 22%–45% for 26 min, 45%–95% for 2 min, 95% for 2 min. Data dependent acquisition (DDA) was performed in profile and positive mode with Orbitrap analyzer at a resolution of 120,000 (@200 m/z) and m/z range of 350–1,600 for MS1; For MS2, the resolution was set to 15,000 with a dynamic first mass. The automatic gain control (AGC) target for MS1 was set to 3E6 with max IT 50 ms, and 1E5 for MS2 with max IT 110 ms. The top 20 of most intense ions were fragmented by HCD with normalized collision energy (NCE) of 27%, and isolation window of 1.2 m/z. The dynamic exclusion time window was 45 s, single charged peaks and peaks with charge exceeding six were excluded from the DDA procedure.

### Proteome Discoverer database search

Proteome Discoverer (PD) software (Version 2.4.0.305) and the built-in Sequest HT search engine were used to process Vendor’s raw MS files. MS spectra lists were searched against their species-level UniProt FASTA databases (uniprot-Mus + musculus-10090-2020-10.fasta). Here, carbamidomethyl (C) as a fixed modification, oxidation (M) and acetyl (N-term) as variable modifications, and trypsin as proteases. A maximum of two missed cleavage(s) was allowed. The threshold of false discovery rate (FDR) was 0.01 for both PSM and peptide levels. Peptide identification was performed with an initial precursor mass deviation of up to 10 ppm and a fragment mass deviation of 0.02 Da. Unique peptide and Razor peptide were used for protein quantification and total peptide amount for normalization. All other parameters were reserved as default.

### Measurement of intracellular ROS

Here, the measurement of intracellular ROS levels were using 2', 7'-dichlorodihydrofluorescein diacetate (H_2_DCF-DA) (Beyotime Biotechnology, Hangzhou, China). When cells are cultured to mid-log phase, 0.6 OD_600_ cells were collected, and then washed with 1×phosphate-buffered saline (PBS) once. The pellets were re-suspended with 500 μl PBS, then added H_2_DCF-DA at a final concentration of 100 μM. The mixture then incubated in darkness for 1 h at 30°C, 100 rpm. After that, cells were collected and washed three times with 1 × PBS. 300 μl 1 × PBS were used to re-suspended the pellets. The mixture then detected by the DCF fluorescence using CytoFLEX FCM flow cytometry (Beckman coulter, CA, United States). The excitation and emission wavelength were set up at 488 nm and 525 nm, respectively. Cells with fluorescence intensities ranging 10^3^–10^5^ were selected and counted, and at least 20,000 events were collected for each sample.

## Results and discussion

### Construction of *Z. mobilis* ZMNP and cell growth under different conditions

**FIGURE 1:**
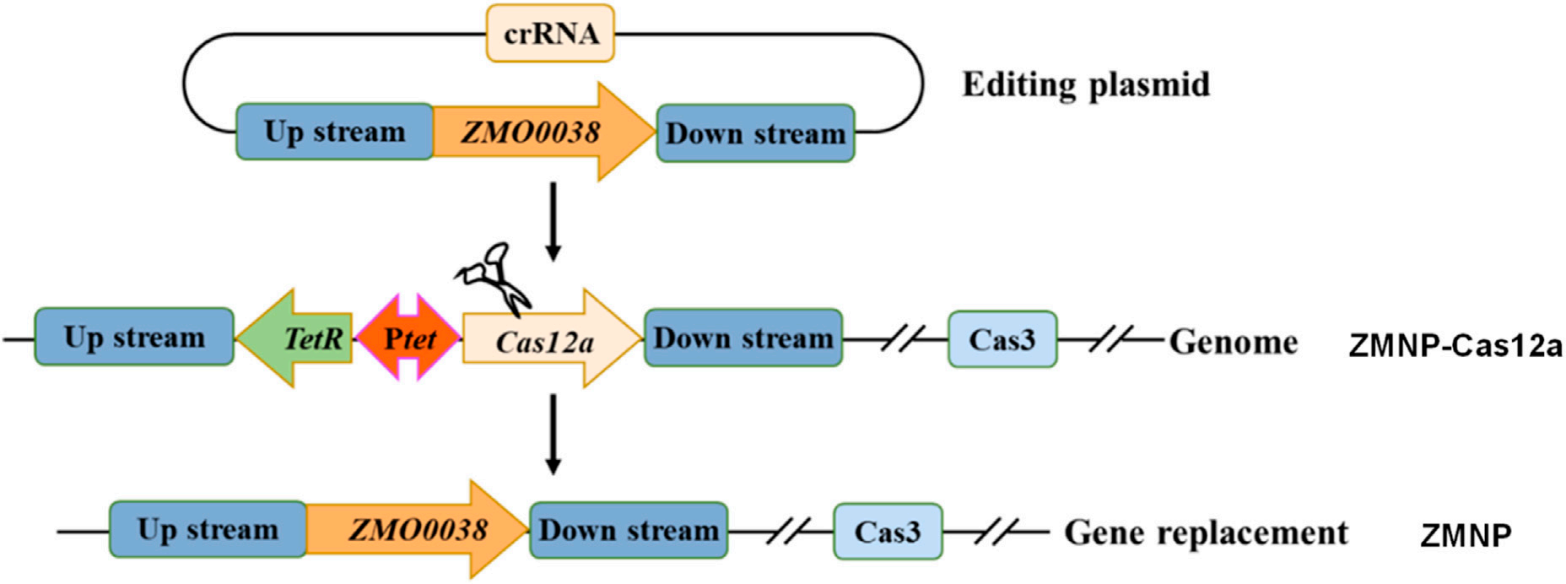
The scheme of ZMNP mutant construction by replacing cassette TetR-P*tet*-*cas12a* with *ZMO0038* in *Z. mobilis* ZMNP-Cas12a using the native type I-F CRISPR system.

We previously constructed a mutant strain ZMNP-Cas12a that contains a cassette of TetR-P*tet*-*cas12a* that replaced *ZMO0038* locating between *ZMO0037* and *ZMO0039*. Compared to wild-type ZM4, ZMNP-Cas12a also lacks four native plasmids that were cured by CRISPR-Cas system. Here, we further constructed the mutant ZMNP with the cassette TetR-P*tet*-*cas12a* replaced with the native *ZMO0038* by native type I-F CRISPR system (Figure 1). The final mutant strain ZMNP only lacks four native plasmids compared to ZM4.

Temperature is one of the common physical factors affecting cell growth and microbial fermentation. Moreover, high-temperature ethanol fermentation has advantages of reduced pollution risk as well as cooling costs making it suitable for large-scale bioethanol fermentation (Li et al., 2021). Substrate is another factor that restricts the cost of microbial fermentation. Corncob is abundant with ca 250 million tons produced each year, which is an excellent cellulosic material for commercial xylose and xylitol production. However, the industrial effluent of xylose mother liquor accompanying the xylose and xylitol production becomes a problem. We evaluated cell growth and fermentation performance of ZM4 and ZMNP under different temperatures and different media including xylose mother liquor.

**FIGURE 2:**
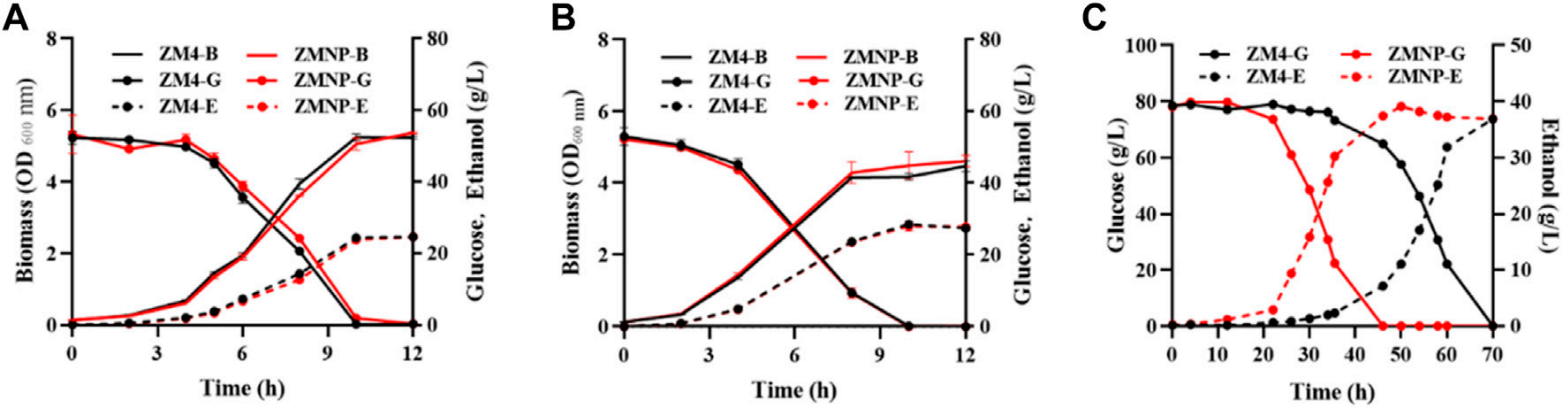
The effect of different temperatures and xylose mother liquor of *Z. mobilis* ZM4 and ZMNP. Growth, glucose consumption and ethanol production of ZMNP and ZM4 under 30°C **(A)** and 40°C **(B)**. Fermentation performance between ZMNP and ZM4 using xylose mother liquor **(C)**. Strains were cultured in 50-ml shake flasks containing 40 ml rich medium RM at 100 rpm. Three replicates were performed for the experiment. B: biomass, G: glucose, E: ethanol.

Cell growth and fermentation performance of ZMNP using glucose under different temperatures or using the xylose mother liquor were compared with ZM4, and the results demonstrated that ZMNP performed similarly to ZM4 using glucose with a growth rate of 0.525 h^−1^ *vs* 0.512 h^−1^ as well as ethanol productivity of 2.44 g/L h^−1^ *vs* 2.38 g/L h^−1^ when cultured under 30°C (Figure 2A), and with a growth rate of 0.395 h^-1^ *vs* 0.399 h^−1^ as well as ethanol productivity of 2.66 g/L h^−1^ *vs* 2.67 g/L h^−1^ when cultured under 40°C (Figure 2B). However, ZMNP did exhibit better glucose consumption and ethanol production than ZM4 when the toxic xylose mother liquor was used. The glucose utilization in ZMNP was 24 h faster than that observed for ZM4, and the maximum ethanol titers of 39.13 g/L and 36.91 g/L were achieved by ZMNP and ZM4, respectively (Figure 2C).

The normal growth and fermentation performance of ZMNP at high temperature and its excellent fermentation performance using xylose mother liquor exhibited that ZMNP could be an ideal chassis for biochemical production from lignocellulosic materials.

### Genetic determinants of ZMNP for enhanced hydrolysate utilization

To determine the potential genetic determinants of ZMNP for increased utilization of xylose mother liquor, next-generation sequencing (NGS) and third-generation sequencing (TGS) technology were applied to identify the potential genetic changes in ZMNP. Combining the data collected from NGS and TGS, we assembled the genome of ZMNP to explore the potential genetic determinants for native plasmid deletion through comparative genomic analysis using the genome of parental strain ZM4 (ATCC 31821) as the reference (Yang et al., 2018b). The genome of ZMNP contains one circular chromosome of 2,058,754 bp only without plasmids that exist in the wild-type ZM4.

The whole-genome resequencing (WGR) results identified a total of five single nucleotide polymorphisms (SNPs) that occurred in open reading frames (ORF) in ZMNP (Table 1 **)**. Among these mutations, two missense mutations were identified in ZMNP located in the coding sequence region of *ZMO0651* (D172N) and *ZMO1733* (S267P), respectively. *ZMO0651* encodes a flagellar hook protein FliD, which is a flagellar cap at the distal end of the flagellar filament protecting the tip of the flagellum (Nedeljkovic et al., 2021). FliD also helps insert flagellin proteins repetitively to grow the flagellar filament (Cho et al., 2019). It was observed that the motility was defective in *fliD* mutants because the flagellin monomers were shed into the outside of cell causing the failure of flagellin polymerization and the form a functional flagellum, revealing that *fliD* was an essential factor in cell motility (Arora et al., 1998; Kim et al., 1999). We simulated the 3D structure of the FliD mutant, and it showed that this missense mutation didn’t cause changes in the 3D structure (data not shown). Therefore, we speculate that this point mutation D172N mayn’t affect its protein function.

**TABLE 1:** Single-nucleotide polymorphisms (SNPs) in ZMNP compared to ZM4.

Locus	Gene	SNP	Frequency (%)	AA change	Product
225216	*ZMO0228*	C→T	100	Synonymous	putative polisoprenol-linked O-antigen translocase
481847	*ZMO0487*	C→T	99.64	Synonymous	HpcH/Hpal aldolase
641565	*ZMO0651*	G→A	99.74	D172N	Flagellar hook protein FliD
934794	*ZMO0915*	C→A	99.88	Synonymous	Copper-translocating P-type ATPase
1780057	*ZMO1733*	T→C	99.51	S267P	Transcriptional regulator OxyR

**FIGURE 3:**
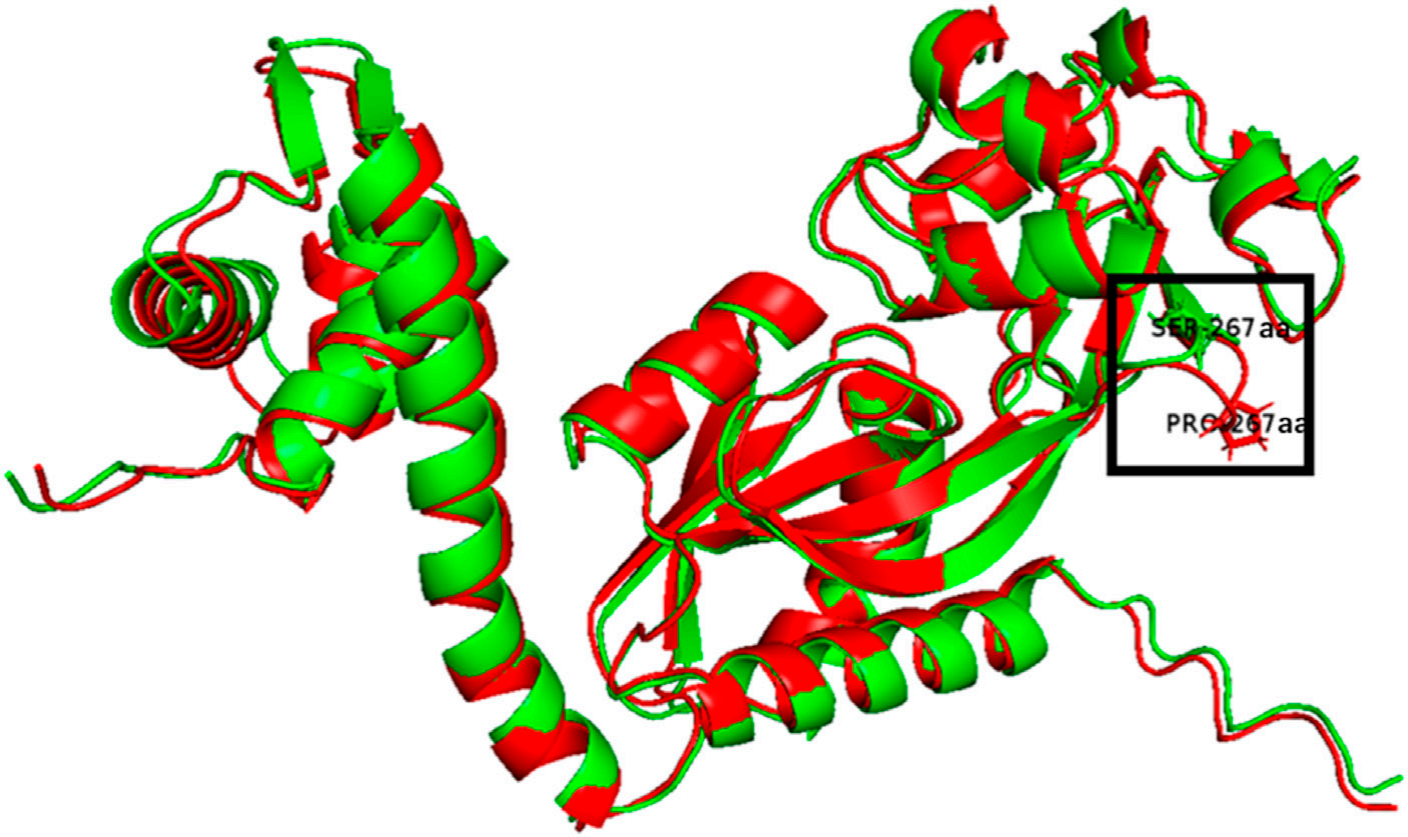
Overlay of the 3D structures of predicted OxyR mutant (Red) and wild-type OxyR protein (Green).

The S267P mutation of OxyR is in LysR-substrate binding domain (IPR005119, 93-295 aa), which probably changes the binding affinity with its substrate (H_2_O_2_) due to the amino acid change from serine to proline, resulting in a protrusion at the 267 aa (Figure 3). Previous research reported that residue 266 aa was an important residue in reduced monomer conformation of OxyR (Anand et al., 2020), and beneficial to stabilize reduced tetramer *via* side-chain interactions (Anand et al., 2020). We speculated that the mutation at the 267 aa might affect the stabilization of the OxyR reduction state, making it more prone to transition to the oxidation state by altering the transcription initiation, and further respond to intracellular oxidative stress signals (Figure 1). This may be one of the reasons that ZMNP is better at utilizing xylose mother liquor.

### Unravel the underlying mechanisms of hydrolysate tolerance of ZMNP through transcriptomic and proteomic studies

#### Overview of quantitative transcriptomics and proteomics

To illustrate the underlying genetic basis of the tolerance to xylose mother liquor in ZMNP, samples of ZMNP and wild-type strain ZM4 cultured at RM and MM were collected for RNA-Seq to explore the global transcriptional differences in ZMNP and ZM4. The differentially expressed genes (DEGs) were identified through analysis of variance (ANOVA) using strains and different media as variables. In addition to the genes in native plasmids, 1020 genes were identified by comparing both strains and media with *p*-value < 0.05 (Supplementary Table S1). Specifically, there were 55 and 87 DEGs comparing ZMNP with ZM4 at RM and MM conditions, respectively, reflecting the difference between strains under different media (Supplementary Table S2). 776 and 813 DEGs were also identified comparing MM with RM conditions of ZM4 and ZMNP, respectively, reflecting the effect of media for different strains (Supplementary Table S3). In addition to genes on native plasmids, 48 DEGs on genome were identified when took the complement of the DEGs under RM and MM media (Supplementary Table S2). Among these significantly DEGs, there were 29 genes upregulated and 19 genes downregulated in ZMNP compared with ZM4 (Supplementary Table S2). All these genes identified between strain comparisons were supposed to influence different functions in cell process, and then further analyzed.

We also used Quantitative proteomics to compare the differences in protein expression of *Z. mobilis* ZM4 and ZMNP in MM medium. We identified 14,801 peptides, which were matched to 1,425 unique proteins (Supplementary Table S4). Differentially expressed proteins (DEPs) which had more than 1.5-fold differences in abundance and less than 0.05 of *p*-value were determined and listed in Supplementary Material, showing the most abundant DEPs with 34 upregulated and 31 downregulated proteins of ZMNP (Supplementary Table S5).

#### Transcriptomic and proteomic profiling of *Z. mobilis* ZMNP compared to ZM4

Gene expression results showed that among the 19 downregulated genes in ZMNP, eight genes (*ZMO0383*, *ZMO0387*, *ZMO0388*, *ZMO0392*, *ZMO0397*, *ZMO0398*, *ZMO0930*, *ZMO1787*) encode hypothetical proteins, four genes encode levansucrase (*ZMO0374*, *ZMO0375*), levansucrase regulator (*ZMO0934*) and glycohydrolase secretase (*ZMO0064*), and two genes encode transporters (*ZMO0916*, *ZMO1457*) (Supplementary Table S2). Among them, the genes encoding levansucrase include *sacB* (*ZMO0374*) that catalyzes sucrose hydrolysis to glucose and fructo-oligosaccharides, and *sacC* (*ZMO0375*) that hydrolyzes sucrose to glucose and fructose (Gunasekaran et al., 1990). *zliE* (*ZMO0934*) encodes levansucrase regulator that stimulates the production of sucrose-hydrolyzing enzymes such as SacB and SacC (Kondo et al., 1994). *zliS* (*ZMO0932*) is a secretion-activating factor that contributes to the secretion of sucrose-hydrolyzing enzymes (Kondo et al., 1994). *oprB1* (*ZMO0064*) encodes a glucose porin that enables glucose entry into the periplasmic space and transports it to the cytoplasm *via* an ABC transport system (del Castillo et al., 2007). The downregulation of these genes indicate that the capacity of glycolysis, transport, and secretion of hydrolyzing enzymes was decreased in ZMNP, especially the hydrolysis of sucrose and the secretion of sucrose-hydrolyzing enzymes. The proteomic profiling results also showed that SacB and ZMO0916 were upregulated (Supplementary Table S5).

**FIGURE 4:**
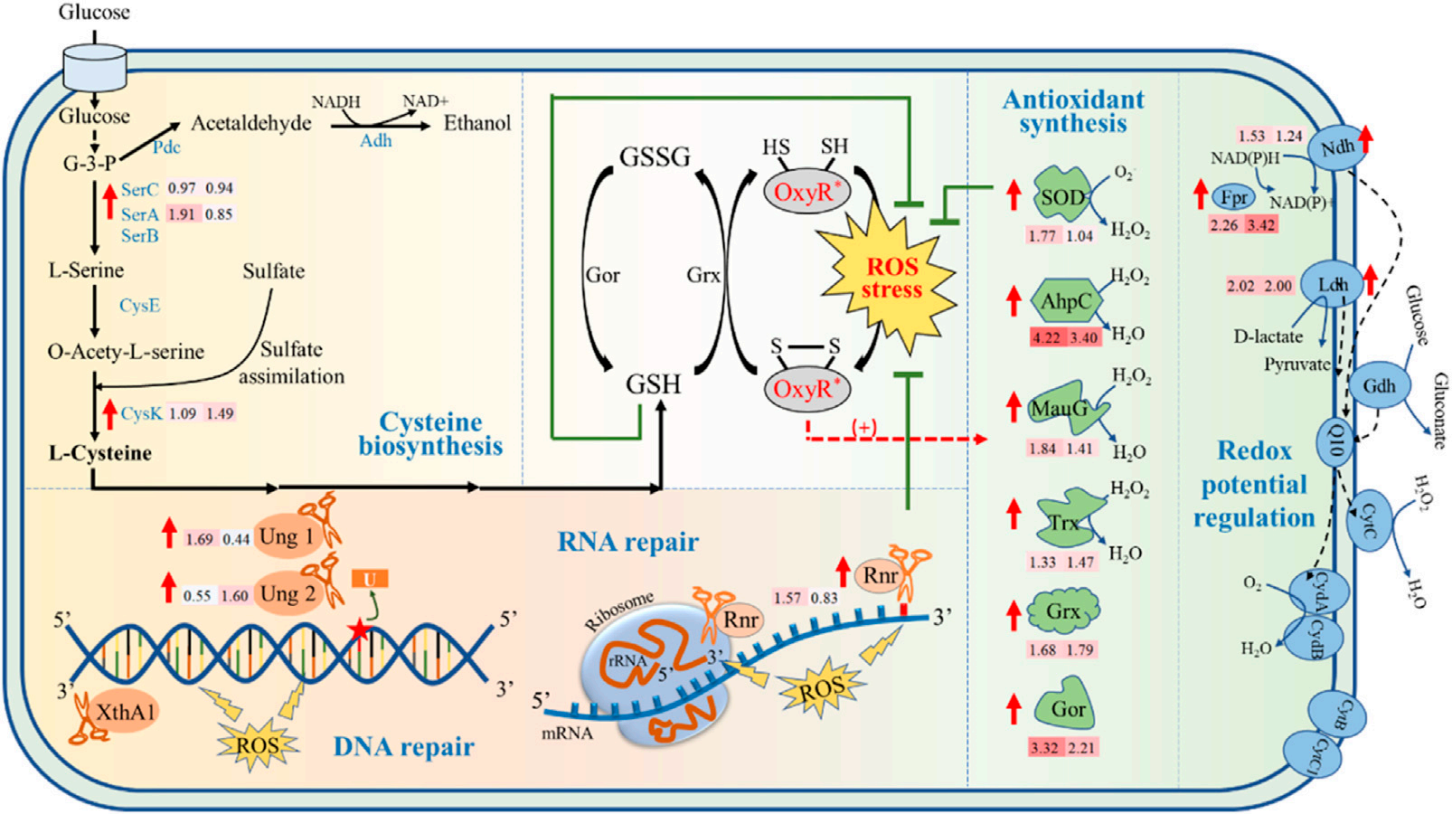
Potential molecular mechanism of inhibitor tolerance of ZMNP. AhpC: Alkyl hydroperoxide reductase, Fpr: Ferredoxin-NADP (+) reductase, Gor: Glutathione reductase, Grx: Glutaredoxin, GSH: Reduced glutathione, GSSG: Oxydized glutathione, G-3-P: Glyceraldehyde 3-Phosphate, Ldh: Lactate dehydrogenase, MauG: Cytochrome-c peroxidase, Ndh: FAD-dependent pyridine nucleotide-disulfide oxidoreductase, Rnr: Ribonuclease R, SOD: Superoxide dismutase, Trx: Thioredoxin, Ung: Uracil-DNA glycosylase, XthA1: Exodeoxyribonuclease III XthA.

In addition, among the 29 upregulated genes in ZMNP, 15 genes were general stress response genes (Figure 4), especially the genes regulated by OxyR. It mainly includes 1) enzymes that are regulated by OxyR for synthesis of cellular antioxidants to remove peroxide; 2) enzymes that regulate and stabilize intracellular redox potential; 3) enzymes that repair DNA and RNA; and 4) enzymes associated with cysteine biosynthesis.

i) Upregulation of cellular antioxidants in ZMNP to remove peroxide for enhanced inhibitor tolerance. In the presence of ROS, the reduced form of OxyR could be transformed into oxidized OxyR by rapid formation of an intramolecular disulfide bond (Jo et al., 2015). In *E. coli*, the oxidized OxyR activates the expression of genes in response to oxidative stress (Ren et al., 2017) such as *sod*, *ahpC*, *yhjA*, *trx*, *grx*, *gor katG*, *ahpF*, and *hemF* (Charoenlap et al., 2005; Hishinuma et al., 2006; Zeller and Klug, 2006). The transition between reduced and oxidized OxyR also involves in the balance of GSH and GSSG, the multifunctional intracellular antioxidants with the reduced form and oxidized form, respectively. The oxidized OxyR is reduce by glutaredoxin (Grx) accompanied by the consumption of GSH and the production of GSSG. And GSSG could be transformed to reduced GSH by glutathione reductase (Gor), thus maintaining the balance between GSH and GSSG.

Our RNA-Seq results also demonstrated that six genes that are regulated by OxyR for oxidative stress response were upregulated. *ZMO1060*, encoding superoxide dismutase (SOD) to convert O_2_^●-^ to O_2_ and H_2_O_2_ (Zhao et al., 2021), was upregulated in ZMNP. This is similar to the results of previous studies that *sod* is positively regulated by OxyR (Zhou et al., 2021) and usually upregulated under stress conditions (Shatalin et al., 2011; Mironov et al., 2017). In addition, *ZMO1732* (*ahpC*), *ZMO1136* (*mauG*), and *ZMO1097* (*trx*) that are participated in the direct removal of H_2_O_2_ were significantly upregulated in ZMNP compared to ZM4. Alkyl hydroperoxide reductase subunit C (AhpC) proteins are the catalytic subunits of alkyl hydroperoxide reductases (Mongkolsuk et al., 2000), which are members of peroxidases to protect against H_2_O_2_. In ZMNP, the expression of *ZMO1732* was 10 folds and 18 folds higher than that in ZM4 when cultured under RM and MM, respectively. Protein MauG encoded by *ZMO1136* was 44% identity to YhjA of *E. coli* (NC_002695.2) by BLASTP analysis. It catalyzes the reduction of H_2_O_2_ to H_2_O. The proteomic profiling results also showed that *ahpC* and *mauG* were upregulated. *ZMO1097* encoding thioredoxin (*trx*)-domain containing protein was also upregulated in ZMNP. Thioredoxin is a key antioxidant system that regulates protein dithiol/disulfide balance through its disulfide reductase activity to combat oxidative stress such as H_2_O_2_ (Lu and Holmgren, 2014). We speculate that *ZMO1097* was involved in the thioredoxin system and the overexpressed thioredoxin improved the Trx system’s capacity to scavenge H_2_O_2_.

Our RNA-Seq results also demonstrated that the expression of *ZMO0753* (*grx*) encoding Grx that catalyzes the reaction of GSH to GSSG was significantly upregulated in both RM and MM in ZMNP. Furthermore, the expression of *ZMO1211* (*gor*) encoding Gor that catalyzes the reaction of GSSG to GSH was also upregulated in both RM and MM in ZMNP. The proteomic profiling results also showed that Grx and Gor were upregulated in ZMNP. The upregulation of both *ZMO0753* and *ZMO1211* indicates that the fast redox reaction in ZMNP contributes to the quick ROS removal. All six genes (*ZMO1060*, *ZMO1732*, *ZMO1136*, *ZMO1097*, *ZMO0753*, and *ZMO1211*) belong to OxyR regulon regulated by OxyR, revealing that the point mutation of OxyR in ZMNP might alter its three-dimensional structure and leads to the upregulation of genes involved in antioxidant regulation.

ii) Maintained intracellular redox potential in ZMNP for enhanced inhibitor tolerance. The microbial aerobic respiratory chain is an important source for cell metabolism to generate energy and to maintain the redox balance *in vivo*, which is related to cell metabolic processes such as ROS production and resistance to oxidative stress. Previous studies have shown that *Z. mobilis* possess a structural respiratory chain consisting mainly of type II NADH dehydrogenase (Ndh, *ZMO1113*), coenzyme Q10, cytochrome bd terminal oxidase (CydAB, *ZMO1571*-*ZMO1572*) (Kalnenieks et al., 2019), and D-lactate dehydrogenase (Ldh, *ZMO0256*) to contribute electrons to the respiratory chain. Our RNA-Seq data showed that *ZMO1113* and *ZMO0256* were upregulated in ZMNP, which accelerated the electron transport and reduced H_2_O_2_ and O_2_ to H_2_O. *ZMO1753* (Ferredoxin) that reduces NADP^+^ to NADPH is also involved in the balance of the oxidation potential inside the cell. And the upregulation of *ZMO1753* in ZMNP could help maintain the intracellular redox potential.
iii) Improved macromolecular repair in ZMNP for enhanced inhibitor tolerance. H_2_O_2_ produces hydroxyl radicals that oxidize the base and ribose parts of DNA, causing a variety of damages (Hutchinson, 1985; Imlay, 2013). Bacteria actively adopt a variety of oxidative stress measures to cope with oxidative stress, such as nucleotide excision, mismatch repair, and DNA double-strand breaks, which are initiated to protect genomic stability. The transcriptomic results showed that four genes associated with base excision repair were upregulated: *ZMO1114* (*ung1*), *ZMO1648* (*ung2*), *ZMO1401*(*xthA1*), and *ZMO1096* (*rnr*). Uracil-DNA glycosylases (Ung) are enzymes that cleave the bond between deoxyribose and mismatched uracil from DNA (Krokan & Bjoras, 2013; Schormann et al., 2014). Two Ung enzyme genes, *ZMO1114* (*ung1*) and *ZMO1648* (*ung2*), were significantly upregulated in ZMNP, helping repair DNA damage caused by oxidative stress. Exonuclease III plays a key role in base excision repair which is a key repair mechanism to neutralize oxidative stress in DNA (Souza et al., 2006). In *Z. mobilis*, *ZMO1401* encodes a 3' to 5' exonuclease (Exonuclease III), and *ZMO1096* encodes a ribonuclease R to degrade RNA in the 3'-5' direction (Abula et al., 2022). Both *ZMO1401* and *ZMO1096* were significantly upregulated in ZMNP. Proteomic profiling results also showed that XthA1 was upregulated. These results suggested that ZMNP can protect DNA and RNA from damages caused by ROS through upregulation of these DNA/RNA repair proteins.
iv) Enhanced cysteine biosynthesis in ZMNP for inhibitor tolerance. Cysteine pool is crucial for microorganisms to defend against inhibitors. For example, cysteine is usually used for protein and GSH biosynthesis to protect cells against the oxidative stress (Hicks and Mullholland, 2018), and cysteine supplementation in the growth media helped reduce the toxicity of furfural and hydrolysates (Miller et al., 2009; Nieves et al., 2011; Yan et al., 2022). Gene expression results showed that three genes associated with cysteine synthesis were upregulated in ZMNP, including *ZMO1685* (*serA1*) and *ZMO1684* (*serC*) for L-serine synthesis, and *ZMO0748* (*cysK*) that could catalyze O-acety-L-serine and the sulfur assimilation product H_2_S to synthesize L-cysteine. The upregulation of genes in cysteine biosynthesis pathway might increase the concentration of cysteine within cells to promotes GSH biosynthesis against ROS.

#### Upregulation of stress response genes in ZMNP helps reduce ROS level

Inhibitors such as furfural and high concentration of ethanol are well known to induce ROS accumulation. For example, furfural could damage various cellular components such as DNA, lipids, and proteins when ROS was accumulated in cells under stress conditions (Allen et al., 2010; Kim & Hahn, 2013). Ethanol is also one of the inhibitors of cell growth and metabolism in *Z. mobilis*. It can affect a wide range of cellular processes, such as DNA replication and recombination, DNA/RNA repair, transcriptional regulation, carbohydrate metabolism, cell wall/membrane biogenesis, terpenoid biosynthesis, respiratory chain, transport, and universal stress response (He et al., 2012a). All these inhibitors could contribute to an increase of intracellular ROS. *Z. mobilis* ZMNP upregulated global stress response genes that could help effectively reduce the intracellular ROS.

**FIGURE 5:**
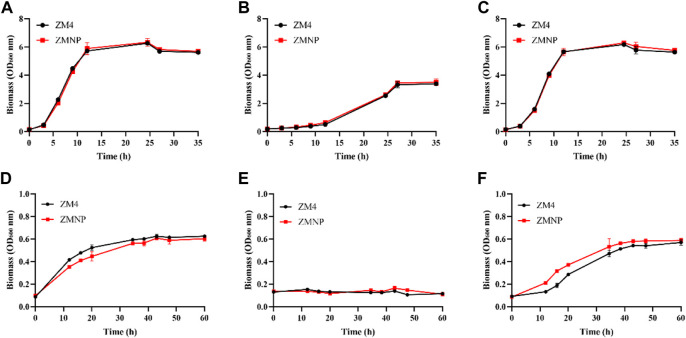
Cell growth of ZM4 and ZMNP responding to different inhibitors. RM **(A)** and RM supplemented with furfural (RMF) **(B)**, or ethanol (RME) **(C)**, as well as MM **(D)** and MM supplemented with furfural (MMF) **(E)**, or ethanol (MME) **(F)** were set up to evaluate the growth of ZM4 and ZMNP. RMF: 2.60 g/L furfural treatment in RM media; RME: 47.36 g/L ethanol treatment in RM media. MMF: 1.25 g/L furfural treatment in MM media, MME: 20 g/L ethanol treatment in MM media. At least two independent experiments were performed with similar results. Values are the mean of one representative experiment with three technical replicates. Error bars represent standard deviations.

To examine whether the expression of genes related to ROS detoxification in ZMNP was upregulated, we determined the growth of ZMNP relative to ZM4 in both RM and MM. The concentrations of furfural that were supplemented into RM and MM were 2.60 g/L and 1.25 g/L, respectively, which were 47.36 g/L and 20 g/L for ethanol supplemented into RM and MM, respectively. Cell growth was similar when ZM4 and ZMNP were cultured in RM or RM media supplemented with furfural or ethanol (Figure 5).

**FIGURE 6:**
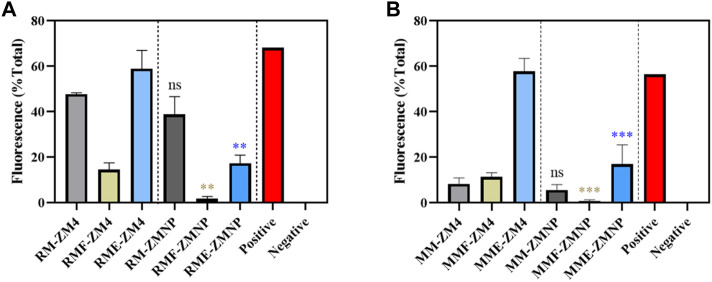
The ROS accumulation of ZM4 and ZMNP responding to different inhibitors. **(A)** ROS accumulation detection under RM with or without inhibitors. **(B)** ROS accumulation detection under MM with or without inhibitors. Data presented in the graphs are the mean ± SD of three replications. T-test analysis was conducted for ROS detection with RM/MM-ZM4 (black asterisk), RMF/MMF-ZM4 (green asterisk) or RME/MME (blue asterisk) condition as the control. ns represents no significant difference (*p*-value > 0.05), * represents a significant difference (0.01< *p*-value < 0.05), ** represents a significant difference (0.001 < *p*-value < 0.01), *** represents a significant difference (*p*-value < 0.001).

Cells cultured in RM, RMF and RME for 3 h, and cultured in MM, MMF and MME for 12 h were collected to detect the ROS levels. The intracellular ROS levels were effectively decreased in ZMNP compared to ZM4 under all culture conditions (Figure 6). Compared with the control strain ZM4 (47.78%, 8.38%), ZMNP (38.87%, 5.70%) exhibited a slight decrease in intracellular ROS under RM and MM media, respectively. The difference of ROS levels between ZMNP and ZM4 were dramatic when cultured in inhibitor-supplemented media. ROS accumulation was detected when ZM4 and ZMNP were cultured in RMF (14.58% *vs* 1.80%) (0.001 < *p*-value < 0.01) and RME (58.97% *vs* 17.33%) (0.001 < *p*-value < 0.01), respectively. And the intracellular ROS levels were effectively decreased in ZMNP compared to ZM4 in MMF (11.44% *vs* 0.76%) (0.001 < *p*-value < 0.01) and MME (57.82% *vs* 16.98%) (0.001 < *p*-value < 0.01). Based on the result, the mechanism of efficient utilization of xylose mother liquor in ZMNP could be due to the overexpression of genes responsive for the general stress and intracellular ROS reduction.

## Conclusion

Robust microorganisms are crucial for biochemical production from lignocellulosic materials. A plasmid-free ZMNP mutant of *Z. mobili*s was constructed in this study, which can efficiently utilize xylose mother liquor. Our multi-omics studies suggested that the S267P mutation of OxyR in ZMNP may help alter the expression of genes associated with global stress response under stress conditions, and ZMNP can reduce its intracellular ROS for efficient lignocellulosic bioethanol production. In addition, the molecular mechanism that OxyR regulates downstream genes to respond to the ROS of *Z. mobilis* proposed in this study can also guide the development of synthetic microbial cell factories for efficient lignocellulosic biochemical production.

## Data Availability

The datasets presented in this study can be found in online repositories. The names of the repository/repositories and accession number(s) can be found in the article/Supplementary Material.

## Abbreviations

AhpC, Alkyl hydroperoxide reductase subunit C; ANOVA, analysis of variance; DEGs, differentially expressed genes; DEPs, differentially expressed proteins; ED, Entner-Doudoroff; Gor, glutathione reductase; Grx, glutaredoxin; GSH, glutathione; GSSG, glutathione (oxidized); LdH, actate dehydrogenase; NdH, NADH dehydrogenase; NGS, next-generation sequencing; OR, open reading frames; PHB, poly-3-hydroxybutyrate; ROS, reactive oxygen species; SNPs, single nucleotide polymorphisms; SOD, superoxide dismutase; TGS, third-generation sequencing; Ung, Uracil-DNA glycosylases; WGR, whole-genome resequencing.
